# Supplementary figures and images for: Investigating the dose-dependency of the midgut escape barrier using a mechanistic model of within-mosquito dengue virus population dynamics
Source: PLoS Pathog. 2024 Apr 1;20(4):e1011975. doi: 10.1371/journal.ppat.1011975 (PMC11008821; doi:10.1371/journal.ppat.1011975)

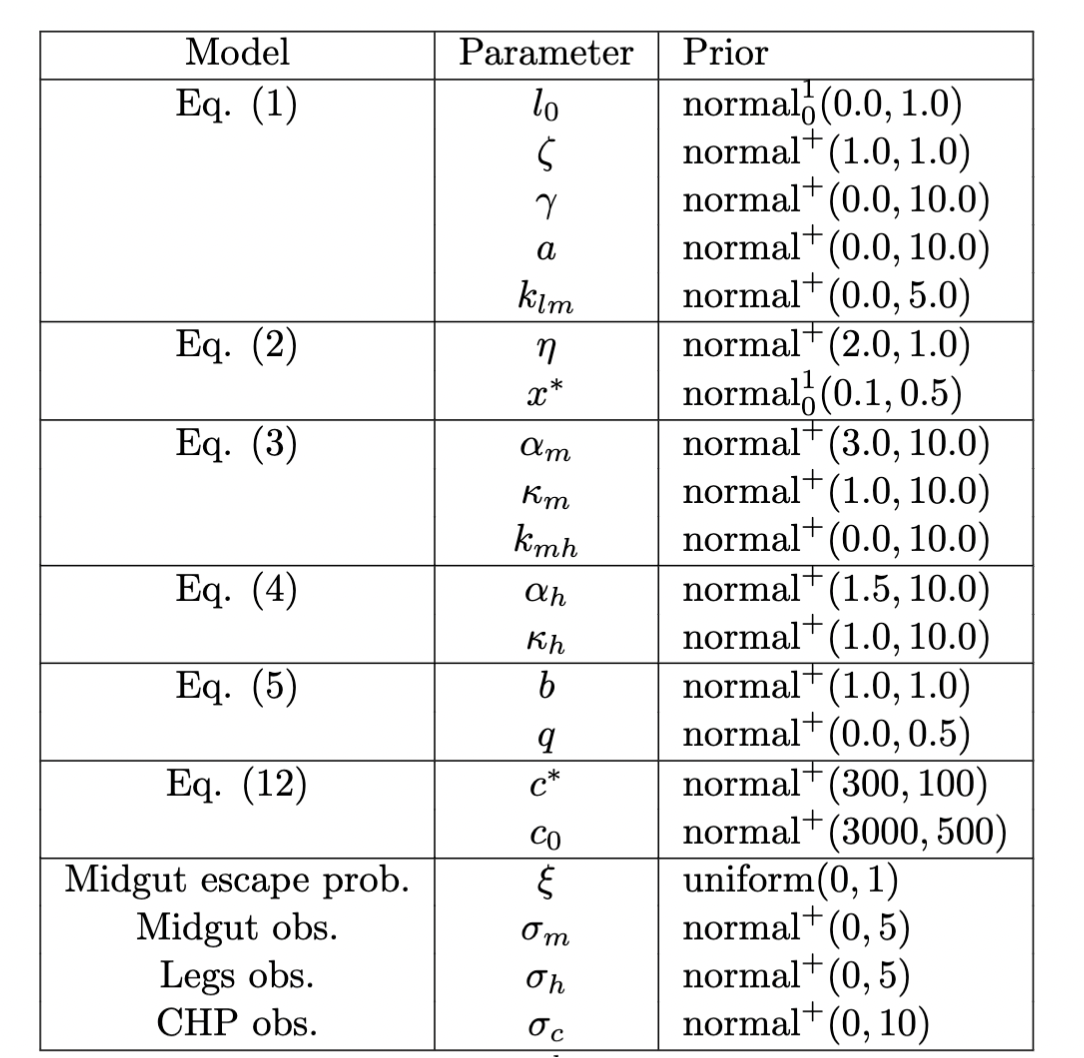

Supplement: S1 Table — Here, distab() indicates a distribution truncated so its domain is between a and b; dist+() denotes a distribution truncated to have support only over positive values. (PNG) [file ppat.1011975.s001.png]

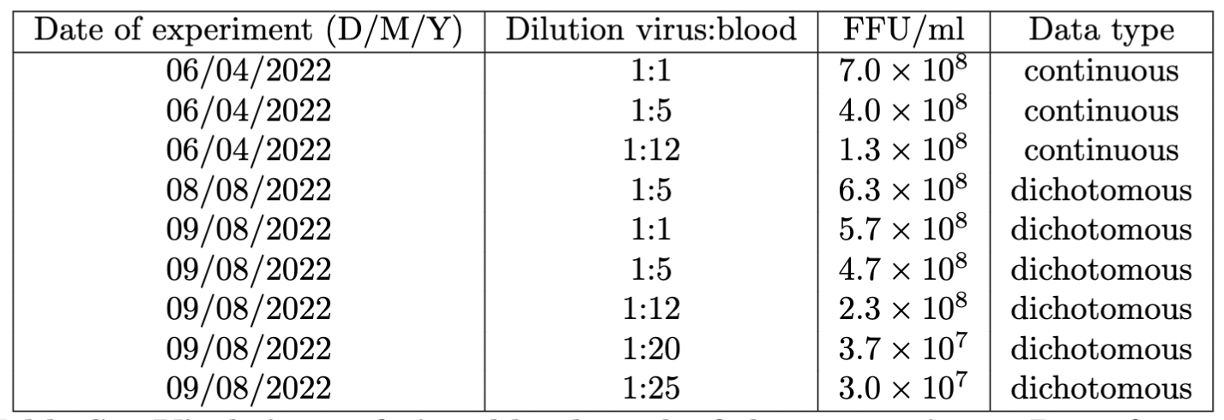

Supplement: S2 Table — Date of experimental feeds and dilution of virus:blood fed to mosquitoes. Viral titers were measured using focus forming assay and viral foci indicating viable viral particles were counted and used to calculate focus forming units per milliliter (FFU/ml). Data type for each experiment indicates whether infection was measured in a dichotomous, positive:negative manner, or whether viral titers were assessed continuously via qPCR. (PNG) [file ppat.1011975.s002.png]

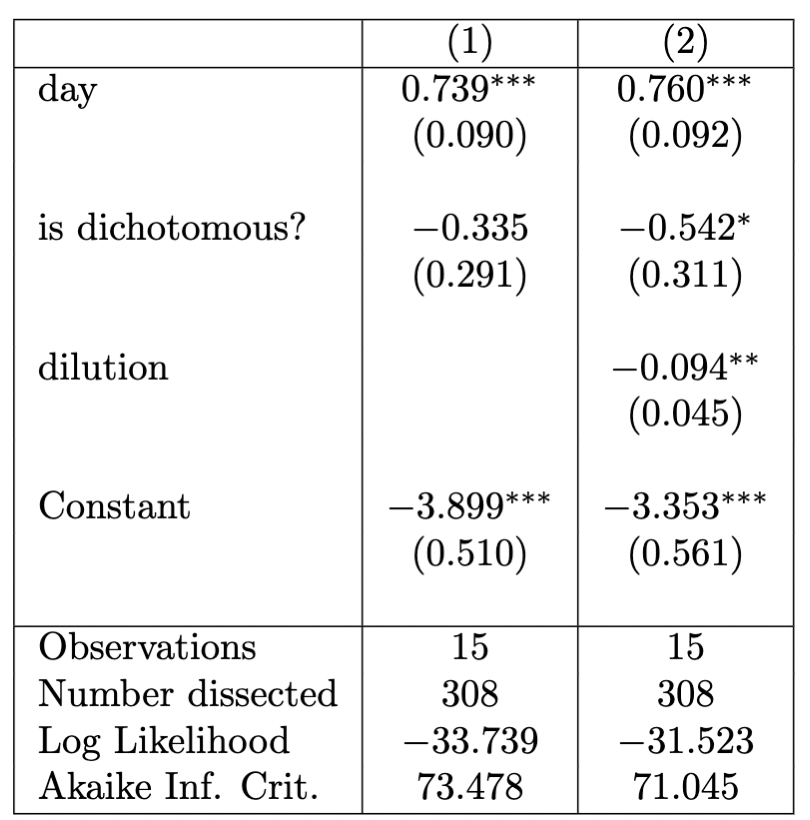

Supplement: S3 Table — In both regressions, a binomial sampling model was assumed with a logistic link function. The logistic model success variable was the number of positive legs in those specimens with infected midguts. Note, *p<0.1; **p<0.05; ***p<0.01. (PNG) [file ppat.1011975.s003.png]

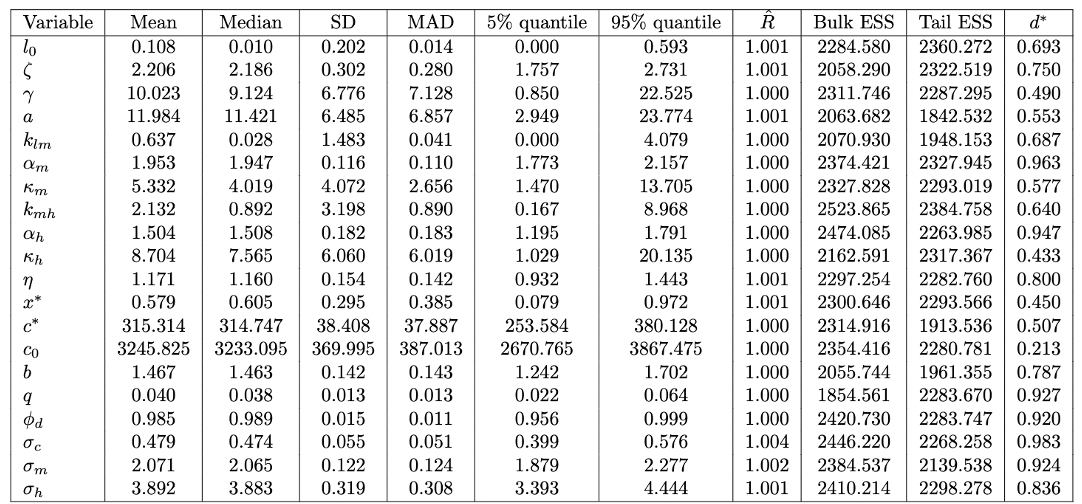

Supplement: S4 Table — The metric d* measures the discrepancy between the (marginal) posterior distribution of a parameter and its prior: a value of 1 indicates that the two distributions have no overlap; a value of 0 indicates that the two distributions are the same. d* is calculated by fitting unidimensional kernel density estimators to each of the two distributions and using these as classifiers (where the class with the highest probability density is the class prediction). d* is computed as 2(A − 0.5), where A is the classification accuracy across an independent test set. The MCMC convergence diagnostics, R^ and the two bulk ESSs, are calculated using the posterior R package [75]. (PNG) [file ppat.1011975.s004.png]

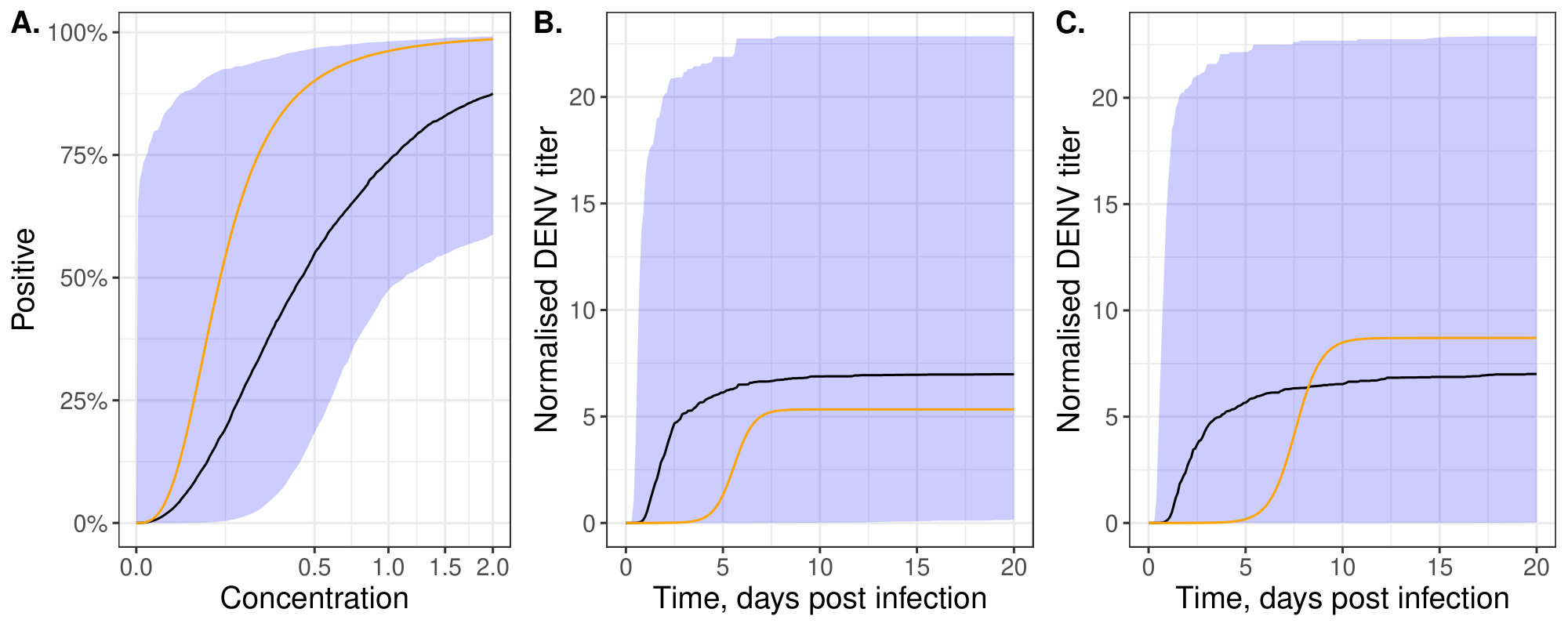

Supplement: S1 Fig — Panel A shows summaries of the prior predictive distribution for ϕ(.) in Eq (5). In panels B and C, we show summaries of the prior predictive distribution for the logistic equation components of Eqs (3) & (4), respectively. Specifically, in these panels, we show the solution of the logistic equation: dy/dt = αy(1 − y/κ) with y(0) = 0.0001 assuming the priors for the midgut (A) and legs (B). In all panels, the summaries were created using 1000 draws from the prior predictive distribution, and the ribbons show the 2.5%-97.5% quantiles and the black lines show the prior median. The orange lines show the posterior median estimates of each quantity. (TIFF) [file ppat.1011975.s005.tiff]

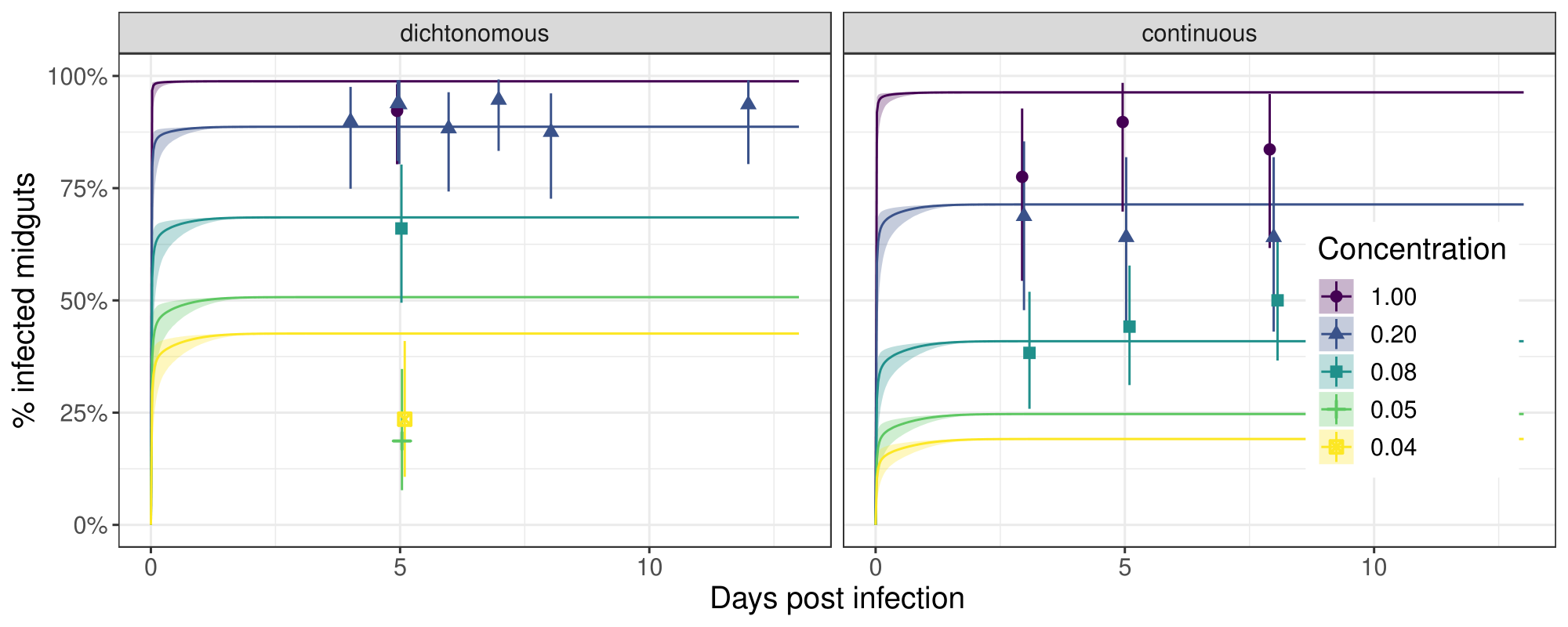

Supplement: S3 Fig — Both panels show the proportion of positive midguts as a function of days post-infected blood meal (DPI) for a range of infectious blood meal doses (coloured lines) relative to our highest “base” dose (concentration = 1; red colouring). The points show the experimental data, and the solid lines show model simulations. The left-hand panel is an exact replicate of Fig 2A and corresponds to the dichotomous experiments; the right-hand panel corresponds to the continuous experiments. The points and whiskers indicate the experimental data, and the line and shaded ribbons represent the model simulation. The solid points indicate the posterior median proportions, and the whiskers indicate the 2.5%-97.5% posterior quantiles, assuming a uniform prior on the proportion. The lines represent the posterior median simulation and the ribbon represents the 2.5%-97.5% posterior quantiles in the median. In both plots, the modelled % infected midguts values were determined by calculating the probability the titer exceeds the detection threshold, specified by Eq (8). (TIFF) [file ppat.1011975.s007.tiff]

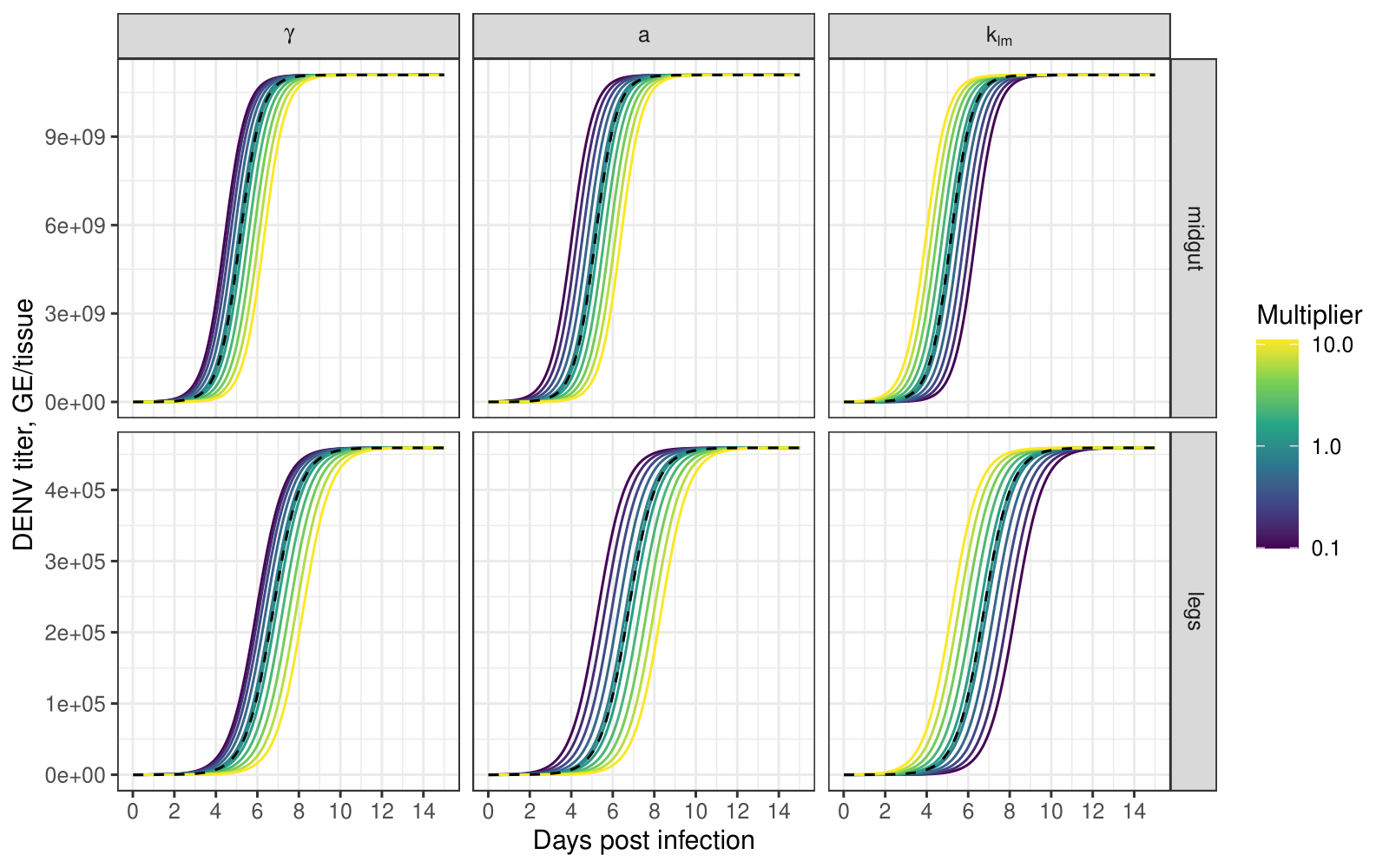

Supplement: S4 Fig — Each column shows the impact of varying a model parameter on the resultant dynamics. The top / bottom rows show the viral dynamics in the midgut / legs. The parameters are varied by multiplying the estimated posterior mean value by a multiplier, shown by the colouring. The black dashed lines shows the model simulations at the posterior mean values. (TIFF) [file ppat.1011975.s008.tiff]

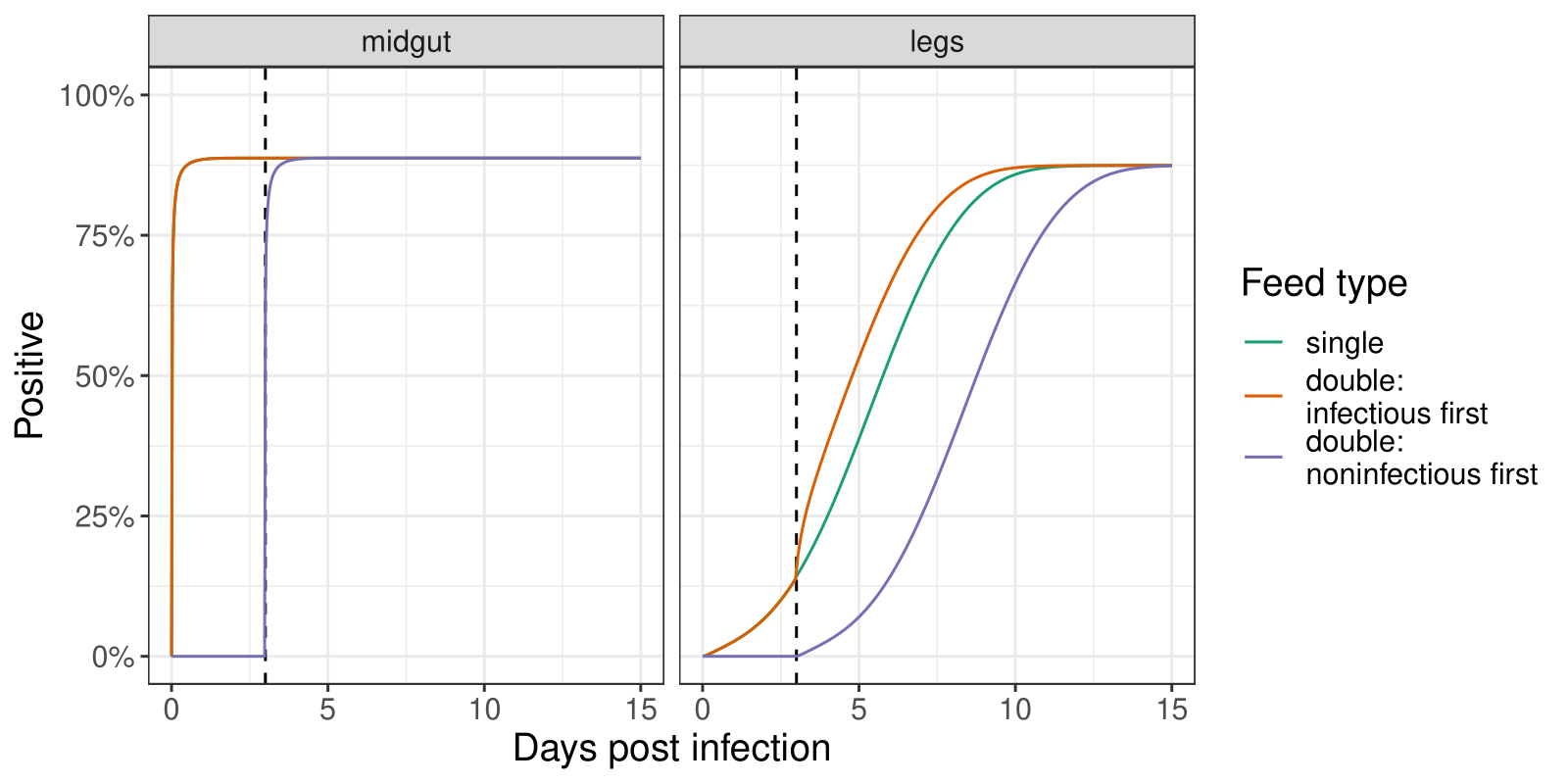

Supplement: S5 Fig — The line colouring shows results for three feed types: mosquitoes fed only at time 0 (“single”); mosquitoes fed an infected blood meal at time 0 followed by an uninfected blood meal 3 DPI (“double: infectious first”); and mosquitoes fed an uninfected blood meal at time 0 followed by an infected blood meal 3 DPI (“double: uninfectious first”). Panel A / B shows the modelled proportion of the midguts / legs positive to infection over time across the three feeds. The dose of initial infection corresponds to a concentration of 0.5 (the same as in Fig 6). In both plots, the modelled % infected values were determined by calculating the probability the titer exceeds the detection threshold. (TIFF) [file ppat.1011975.s009.tiff]

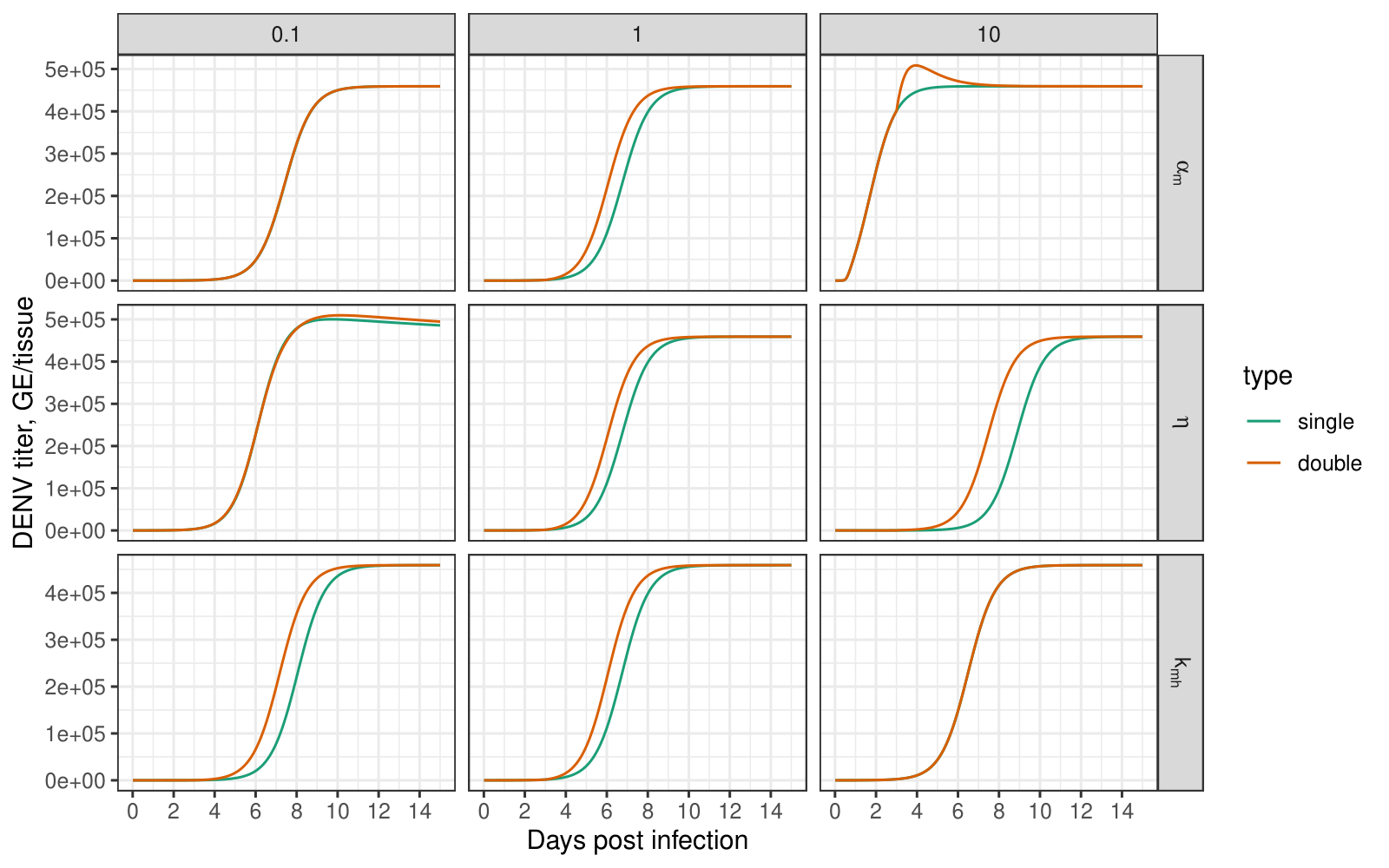

Supplement: S6 Fig — Each row corresponds to a parameter; each column to a multiple of the posterior mean of that parameter. The lines show the model-predicted infection profiles for mosquitoes that were either singly fed or those fed an infectious blood meal at 0DPI, followed by a second noninfectious blood meal at 3DPI. Each panel corresponds to model predictions for a single parameter being varied in isolation. (TIFF) [file ppat.1011975.s010.tiff]

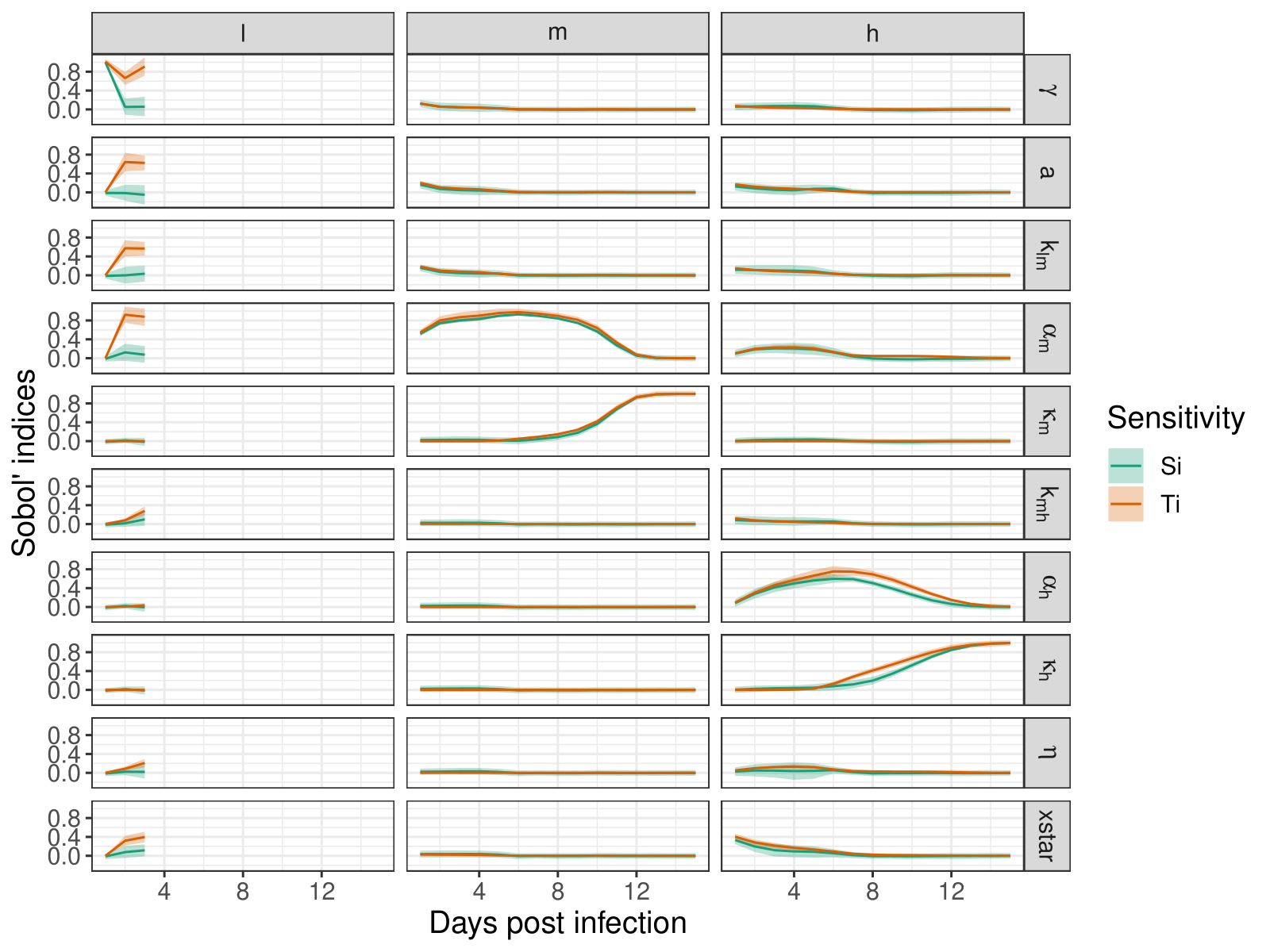

Supplement: S7 Fig — Each column shows the modelled output considered; each row shows results corresponding to the labelled parameter. The coloured lines correspond to the first order (‘Si‘) and total order (‘Ti‘) Sobol’ indices, and the ribbons show the 95% confidence intervals in these quantities as calculated via the sensobol R package [76]. Sensitivities were estimated using 210 model simulations sampled across a subset of parameter space using Quasi-Random numbers (the default package option). The subset of parameter space considered was the hypercube bounded below at 2/3 times the posterior median estimates and above at 3/2 times these estimates. Note, the level of virus in the lumen decays towards zero in the first three days following a blood feed (here mosquitoes were singly fed), so we remove the subsequent sensitivities from the plot to avoid error due to numerical underflow. (TIFF) [file ppat.1011975.s011.tiff]

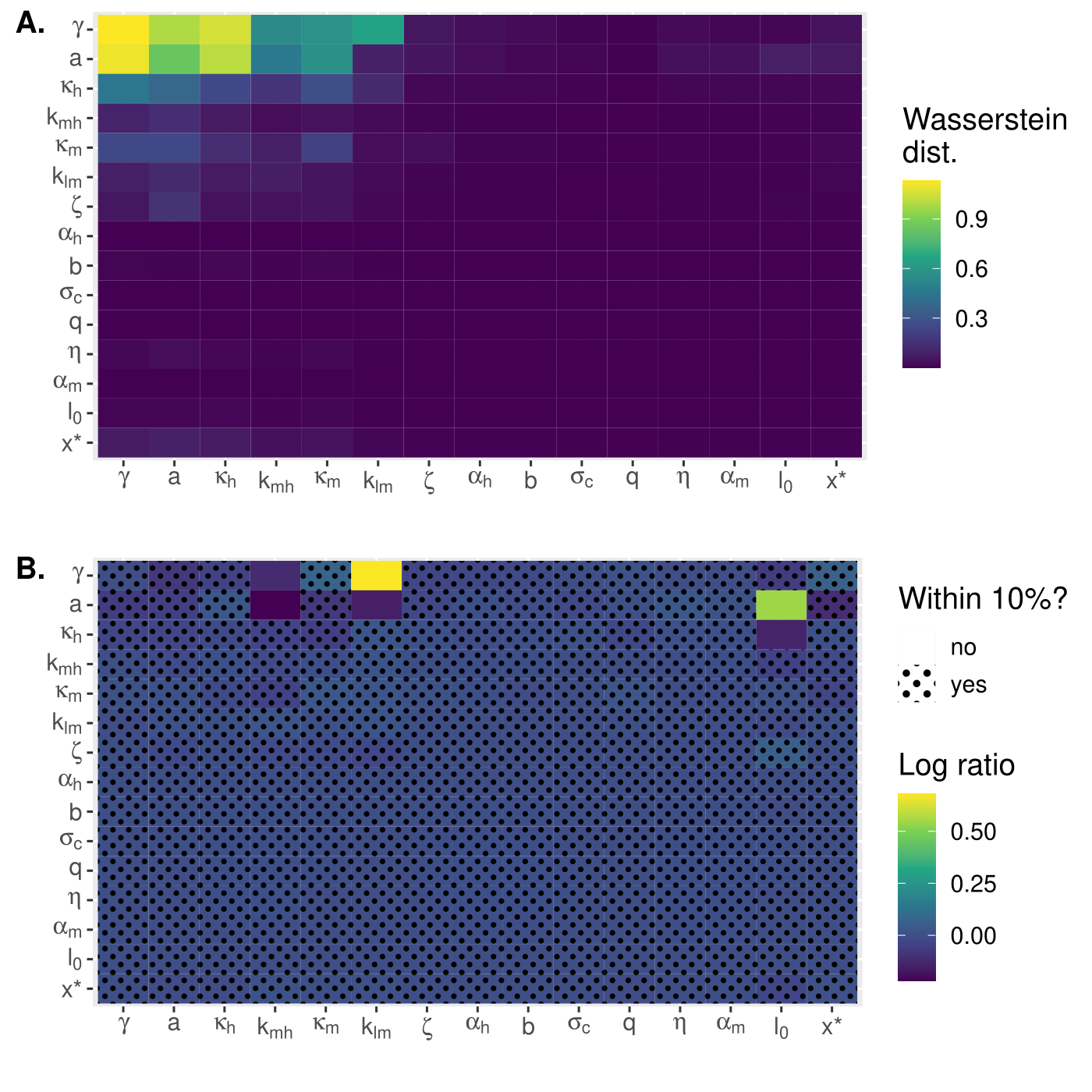

Supplement: S8 Fig — The vertical axis displays the parameter whose prior was perturbed by multiplying the scale parameter by a factor of 5: for example, a ∼ normal(0, 10) becomes a ∼ normal(0, 50). Within a given row, the colouring shows the effect of these changes to the prior on the posterior distributions, as quantified by (A) the Wasserstein distance and (B) the ratio of the posterior mean estimates between the posterior distribution estimated using the priors shown in Table S1 Table and the perturbed ones. The perturbed posteriors were estimated using Pareto-smoothed importance sampling via the adjustr package [77]. In (B), the patterning indicates those cases where the posterior mean from the perturbed priors was within 10% of the original estimate. The rows have been ordered according to the Pareto-k value (larger values at the top). Note that the first three rows (kmh: γ) have Pareto-k values above 0.7, indicating unreliable estimates [78]. (TIFF) [file ppat.1011975.s012.tiff]
