# Supplementary material for: Investigating the dose-dependency of the midgut escape barrier using a mechanistic model of within-mosquito dengue virus population dynamics
Source: PLoS Pathog. 2024 Apr 1;20(4):e1011975. doi: 10.1371/journal.ppat.1011975 (PMC11008821; doi:10.1371/journal.ppat.1011975)
Supplement: Attachment — Submitted filename: ReviewComments.docx [file ppat.1011975.s013.docx]

This paper develops a deterministic model of within-mosquito viral dynamics. To be able to link this deterministic model to results from vector competence experiments, it is assumed that there is some probability of midgut infection which follows a logistic function, and it is assumed that there is some viral titre measurement error. Overall, the paper is well written and well communicated to appeal to both those carrying out vector competence experiments and those interested in modelling viral dynamics. I would highlight that I can comment on the biology/ maths interface, but I am not a mathematician, so I have not commented on any nuances of the maths.

I don’t have any major concerns about the paper but would suggest the comments below are addressed before being considered for publication.

**Introduction**

It is well reported that midgut infection is initiated by fewer than *c.* 10 virions and in our recently published paper we develop a stochastic model of within-mosquito viral dynamics (<https://journals.plos.org/ploscompbiol/article?id=10.1371/journal.pcbi.1011520>). This inherently stochastic process can be shown to explain the dose-response of the probability of midgut infection and the variation between mosquitoes in the response of time to infection. While of course I would suggest you refer to this article because it’s mine 😊, I do think it would be great to compare/ contrast the two approaches (nice that we must have been working on this at the same time from different perspectives).

General: I’d like to know why you didn’t go for a stochastic approach in the first place? In addition, why you didn’t mechanistically model cell infections which may have led to the model having a greater number of more biologically meaningful parameters (see comment on Line 76)?

Line 48: ‘*With the exception of a single study (21) measurements of dissemination represent the proportion of all blood-fed mosquitoes with disseminated infection*’ but in (22) Lequime et al (2020) they also report that ‘*systemic infection prevalences were measured by counting the number of mosquitoes with infected heads over the number of mosquitoes with an infected body*’ and Fig. 4 in this paper demonstrates the effect of virus dose and time on the proportion of infected mosquitoes with disseminated infection. So, there are actually two studies that do show the results in this way.

Line 49: In (21) - Tesla et al (2018), in the results it states in the results in section ‘*The effect of viral dose on ZIKV transmission efficiency’ ‘we did identify a significant interaction between ZIKV dose and days post-infection for both response variables (dissemination and transmission efficiency). This interaction demonstrates that variation in viral dose significantly affects the rate at which virus escapes the midgut and salivary gland barriers…’* Therefore, please remove ‘*with small sample size’* on line 49 as I don’t see how this is relevant, given they found a significant interaction between dose and time.

I would also suggest that it is possible from other studies to get the proportion of infected mosquitoes with disseminated infection just by swapping the denominator; it’s just not usually how these results are presented aside from in references 21 and 22. I think the more pertinent thing to highlight is perhaps how few studies carry out at least three virus doses and at least three time points to obtain data sufficient to quantity dose-time effects on probability of dissemination?

Line 76: mechanistic model – only parts of it are mechanistic right? – it might be good to justify in the introduction or methods why you didn’t explicitly model virus-cell interactions?

Line 78: ‘*Our results suggest that higher infectious doses hasten the rate at which mosquitoes become infectious’*. This has already been shown in 21 and 22, so perhaps frame the last paragraph of the introduction as a set of aims instead which explicitly shows how this study adds to the knowledge generated in 21 and 22? How is this study different? Does the mechanistic approach genuinely contribute more insight than statistical approaches?

Line 79: ‘*Our model also reveals a mechanism by which an initial infectious blood meal followed by a non-infectious blood meal can increase this rate, a characteristic of the experimental data we collected*…’. Again, unless this is the format requested by the journal, I think it would be better to outline the aims of the study. Here, after reading the method, I am not sure that the model ‘reveals’? It more appears that you have observed this phenomenon then developed a model that incorporates this with a term that accounts for slower rates with time post bloodmeal(s)?

**Methods**

Line 180: What is the evidence that there is some maximum invasion rate?

Line 186: What is the evidence that the stretching of the midgut influences the rate at which virions invade midgut epithelial cells?

Line 228: without time series for individual specimens – but you do have how titer differs between specimens at a single time point and also the data on proportion infected by dose and time?

Line 229: Measurement of what?

Line 230 to 234: Why were these methods not used in the current study?

Line 250/ 314: With reference to S1A: this seems to indicate that the functions wouldn’t go to zero? Can you explain in the legend what ‘concentration’ represents – can this be related back to actual PFU/ml in experiments? It also seems that the function assumes that there will never be all mosquitoes infected irrespective of the virus concentration of the bloodmeal?

Lines 249 – 268: For non-mathematicians would it be possible to explain in lay terms here how the logistic function is used with the ODE model to get results like that shown in Fig 2A – how you get variation in % midguts infected over time? It’s not clear to me, so perhaps it’s not clear to others.

**Results**

Line 358 – would be better to be quantitative rather than ‘rapidly’ – is this seconds/ minutes/ hours/ days?

Line 364 – here it says that the first time point you have data for is 3 DPI so how can any assumptions be made about what is happening prior to this time point? Most of the blood meal will have been digested by day 3, therefore I would expect very little virus to be left in the luman after this point. Suggestion of infection prior to 3 DPI isn’t really rapid.

Line 369 – and Fig2A – as per my comment in the method, because I’m not quite clear how the logistic function is linked to the ODE model, I can’t understand what is determining the rapid increase, then plateau of % of infected midguts, could there also not be a model structure where there is a much slower rise in the first 3 days? You don’t have data to support what is happening prior to 3 dpi. In the least perhaps in the Discussion describe what aspects of the model determine these early events and highlight the need for data from earlier time points to inform.

There are some lines that are better off in the Introduction or Discussion e.g. line 361, 368-372, 385-393, 407 – 412, 426 – 429, 432 – 437, 462 – 467, 496 - 509. A lot of these read more like discussion, so please just report your results here and leave comparison with other studies to the discussion section. This will make the results a lot clearer

Line 381: Rather than describing what the plot is please describe the results in the main text and just refer to the figure.

Line 381 and Fig 2B: it looks like the fitted model plateaus at c. 25% below a concentration of 0.03 – this to me doesn’t make biological sense and indeed doesn’t seem to fit well to the lowest two virus concentrations. Can you explain why it plateaus here rather than going to zero as virus concentration declines to zero?

Line 422: ‘Faster growth in the level of virus’ – do you mean: i) in the midgut or other tissues; and ii) higher virus replication rate or earlier escape leads to earlier replication in other tissues which leads to higher viral titres?

Line 423: I don’t see how the earlier detection of dissemination leads to less time required for dissemination for higher initial doses – e.g. this is not a consequence of what is detailed in the prior sentence.

Line 425: ‘…extrapolating…’ – is this not a benefit of a mechanistic approach that extrapolation should give something insightful, even if we have to keep model assumptions and alternative models in mind (as per the findings in Fig 2A)?

Line 430: Rather than ‘mutations’ I would state the parameters in your model that the midgut and dissemination rates are sensitive to in the subtitle. I also guess you mean ‘mutations affecting’. By doing this you also get away from stating the obvious – of course midgut invasion will be sensitive to mutations that affect midgut colonisation; this is a circular argument.

Line 439: you say flavivirus here, but CHIKV is an alphavirus – why not just mosquito-borne virus?

Lines 438 – 446: This should probably go in the methods.

Lines 447 – 449: Just state what the results are rather than stating what you plot (e.g. something like ‘*Simulations (Fig S4) show that a two-order*…’

**Discussion**

Line 554 – 556: ‘crucial to gauge transmission intensity’ – here if you mean force of infection during an outbreak, I suggest that this is not the case, this can be determined from age-seroprevalence curves, measuring cases per unit time etc. In addition, ‘develop effective public health interventions’ is also too generic – I would argue it’s not essential for human vaccine development or necessarily vector control. Can you be more specific here.

Line 561: I would stick to describing the model parameters rather than talking about mutations here and be more specific.

Line 562: ‘series of models at different scales’ – can you be more specific about what you have in mind here? Do you mean coupling within-mosquito with population-level or within-cell and within-mosquito? Without more specific detail the statement is a bit empty.

Line 577: I would suggest that they don’t necessarily avoid the bottlenecks, indeed there is some evidence that they may be advantageous (see discussion in our Plos Comp Bio paper).

Line 659: I would omit this sentence as I don’t think it’s necessary and you are assuming that biologists don’t think quantitatively. This is way too generalising of biologists!
