# Supplementary material for: Investigating the dose-dependency of the midgut escape barrier using a mechanistic model of within-mosquito dengue virus population dynamics
Source: PLoS Pathog. 2024 Apr 1;20(4):e1011975. doi: 10.1371/journal.ppat.1011975 (PMC11008821; doi:10.1371/journal.ppat.1011975)
Supplement: Attachment — Submitted filename: plos_path_response_final.docx [file ppat.1011975.s014.docx]

Dear Editors,

We thank you for your handling of this manuscript and the reviewers for their extremely useful comments, which have allowed us to substantially improve our manuscript.

The main changes we have made to the manuscript are the following:

1. Following a comment from reviewer 2, we have changed the logistic function used to model the probability of successful infection of the midgut so that the model behaves more mechanistically; for example, for a sufficiently high infection load, our model now predicts successful infection to occur in all mosquitoes;
2. Following a comment from reviewer 3, we have changed the Hill function governing the rate at which virus can invade the midgut, again to make the model more mechanistic.

Points 1 and 2 above required us to change a few of our priors and re-simulate our outputs. These methodological changes have, however, made only minor (i.e. quantitative rather than qualitative) changes to our results. Additionally, reviewer 3 asked for changes to figure styling, and we have made additional cosmetic changes to all figures except figure 1.

In reviewing our manuscript, we noticed a mistake in Table S1, where we inadvertently swapped the priors for c0 and c*; we have corrected this. We also noticed that there was a small mistake in the y-axis labels to do with the units of viral titers (previously 'GE/ul' changed to 'GE/tissue'), and we have changed this wherever this appears in the figures. There was also a typo with “data” being referred to as singular which we have corrected.

Below, we respond to the reviewer comments. Alongside this letter, we also make available two versions of our new manuscript: one where the changes are highlighted in orange; another without this colour highlighting.

We hope that you will find our new manuscript suitable for publication and thank you again for considering it. We additionally want to highlight that, should this article be accepted for publication, we would at that point create a release of our software and an associated DOI and hope that it would be ok to include this information during the proofing stage.

Yours sincerely,

Ben (on behalf of coauthors)

**Reviewer #1**
This paper looks at dose dependence of within-mosquito DENV kinetics through a combination of experimental and mathematical work. The manuscript addresses a gap in knowledge regarding this topic, and both the experimental and computational experiments support their overall conclusions. However, there are some minor concerns regarding the organization of the output.

*We thank the reviewer for their comments and are delighted that you found our study worthwhile.*

**Major issues**

None

**Minor issues**

Introduction/Discussion:

See Christofferson et al. “Characterizing the likelihood of dengue emergence and detection in naïve populations” for an example of DENV dose-dependence and transmission.

*Thank you for pointing us to this useful additional study which we now cite in our introduction.*

See Veronesi et al. “Estimating the Impact of Consecutive Blood Meals on Vector Competence of Aedes albopictus for Chikungunya Virus” for additional observations of a second blood meal not affecting CHIKV.

*Thank you for this reference. Veronesi et al. finds there was no increase in dissemination after a second blood meal; however, there were a few things we did not fully understand from their analysis. Their midgut infection rates were quite different between the single-feed and double-feed groups, which is possible but we were unsure why this might be. Additionally, in their calculations, the authors divided the total number of disseminated mosquitoes by the total number tested. When we re-analysed their raw data, using as denominators the number infected, we found that there was an increase in dissemination in the double feed group at day 7 which was lost by day 10, actually in support of the double feed hypothesis.*

M&M:

“… this provides semi-continuous data…” Three data points are longitudinal, but was a curve fit to these data to achieve semi-continuous?

*We thank the reviewer for this astute comment; what we had written previously was ambiguous. It is not the time dimension that was continuous; rather it was the measurement of titer. We have added the following text to our methods to clarify this (previously opaque) statement:*

*“The dissections provided titer data across the time course of the experiment; these data were either continuous and represented viral titers if the measured levels were above the limits of detection, or were zeros and represented measurements below this threshold. Due to this thresholding behaviour, we collectively refer to these data as being ``semi-continuous''.”*

What was the titer of the initial bloodmeal so that the dilutions have a titer associated with them? This is not clear. If this does address this point, consider moving this above.

*Thank you. We measured this with a focus-forming assay and list these titers in Table S2.*

Page 7: Mayton et al. “A method for repeated, longitudinal…” might also be relevant.

*This is really useful; many thanks. We have amended a sentence in our discussion (which examines single mosquito longitudinal measurements) to read:*

*“Alternative approaches which allow for a measure of individual-mosquito level infection dynamics by monitoring the level of virus in mosquito excreta or saliva that is expectorated during bloodfeeding show promise and further work is necessary to compare these techniques with the results obtained by dissection [Mayton et al. 2021…]”*

Results:

“At 3 DPI, mosquitoes feeding on the 1:1 viral dilution show higher midgut DENV titers but this early lead is transient, and the viral titer in the midgut layer is largely independent of the initial blood meal dose by 8 DPI.” – provide a statistical test for this statement.

*This is a really useful comment. We have now added the following statement which clarifies and makes precise our original statement:*

*“At 3 DPI, mosquitoes feeding on the higher viral dilutions showed higher midgut DENV titers than the 1:12 group (Kruskal-Wallis rank sum test performed since data were non-normally distributed: 1:1 versus 1:12, chi^2=12.8, df=1, p<0.01; and 1:5 versus 1:12, chi^2=8.9, df=1, p<0.01; the means for the 1:1 versus 1:5 titers were not significantly different) but this early lead is transient, and the viral titer in the midgut layer does not strongly depend on the initial blood meal dose by 8 DPI.”*

In text, the reference to dashed lines for Figure 5 is a bit confusing, consider saying “within the vertical dashed lines”

*Thank you. We have changed this reference to the following:*

*“However, both the low and high ends of this plot involve extrapolating the model beyond the range of the data (i.e. outside the dashed lines in Figure 5C).”*

Line 435-437 – this paper shows a single timepoint (not the latest) at which dissemination was higher in Ae. albopictus, so “substantially” may not be warranted.

*Thank you for this clarification. We have removed this word so that the sentence now reads:*

*“Mutations in the CHIKV envelope protein have been shown to increase midgut infectivity and rates of dissemination in Aedes albopictus mosquitoes and have been implicated as a cause of outbreaks of chikungunya virus in Reunion island in 2005-2006 (Tsetsarkin et al. 2007) and later in Southern India (Agarwal et al. 2016).”*

Lines 430-431 – this section makes a lot of supposition about mutations. Other external forces affect EIP and vector competence, including temperature which might affect mosquito metabolism rather than viral kinetics directly… Here you are modeling a phenotype, there is no evidence to suggest the A226V mutation affects any of the parameters being toggled here. That’s not to say that this computational experiment is not interesting or informative, but I worry putting it into an assumed mechanism from a mutation will be overinterpreted and mis-cited in future. Consider softening the association with mutations and just put it in the context of alternative phenotypes.

*We thank the reviewer for this comment, and you are right that mutations are not the only mechanism which could influence the parameters we investigate here. We have now modified the subtitle to de-emphasise the association between our work and mutations:*

*“Midgut invasion and dissemination rates are sensitive to fluctuations in parameters representing viral establishment and less sensitive to changes in the rate of viral digestion in the lumen”.*

*We have also added the following text to the bottom of the first paragraph in this section:*

*“Changes in temperature have also been shown to affect the EIP for a number of mosquito-borne viruses (Lambrechts et al. 2011; Tjaden et al. 2013; Winokur et al. 2020), and it is possible that midgut infection and dissemination rates are perturbed by such environmental fluctuations.”*

**Reviewer #2**

This paper develops a deterministic model of within-mosquito viral dynamics. To be able to link this deterministic model to results from vector competence experiments, it is assumed that there is some probability of midgut infection which follows a logistic function, and it is assumed that there is some viral titre measurement error. Overall, the paper is well written and well communicated to appeal to both those carrying out vector competence experiments and those interested in modelling viral dynamics. I would highlight that I can comment on the biology/ maths interface, but I am not a mathematician, so I have not commented on any nuances of the maths.

*We are glad to hear that the reviewer finds our paper well-written and that it walks the fine line between language appealing to both biologists and mathematicians – this is undoubtedly due to the interdisciplinary team (of biologists and mathematicians) who worked on this.*

**Major comments**

I don’t have any major concerns about the paper but would suggest the comments below are addressed before being considered for publication.

**Minor comments**

Introduction:

It is well reported that midgut infection is initiated by fewer than *c.* 10 virions and in our recently published paper we develop a stochastic model of within-mosquito viral dynamics (<https://journals.plos.org/ploscompbiol/article?id=10.1371/journal.pcbi.1011520>). This inherently stochastic process can be shown to explain the dose-response of the probability of midgut infection and the variation between mosquitoes in the response of time to infection. While of course I would suggest you refer to this article because it’s mine 😊, I do think it would be great to compare/ contrast the two approaches (nice that we must have been working on this at the same time from different perspectives).

*It is quite a coincidence that two different groups were working on this simultaneously! And we thank the reviewer for their candour about being one of the study authors. Your excellent study has helped us to better frame this work and also to guide us in developing future models (as we describe in our discussion).*

*We have now amended sections of the manuscript to reference this work and use it to inform our future work. This includes:*

*In methods:*

*“They have also been extensively used to explore within-host pathogen dynamics (...Lord and Bonsall 2023). Despite this work exploring within-host pathogen dynamics, only a limited number of mathematical models have explored viral dynamics in the mosquito (...Lord and Bonsall 2023), and the majority of existing within-vector modelling work has so far focused on malarial parasites…”*

*“Despite being relatively simple, our model provides quantitative predictions which are consistent with our experimental data about how increasing the infection dose influences the rate at which mosquitoes become infectious -- a phenomenon that has been demonstrated in previous experimental studies (Tesla et al. 2018; Lequieme et al. 2020) and modelling work (Lord and Bonsall 2023).”*

*In results:*

*“Our results also indicate that, if more virus is ingested in the blood meal, there is a higher probability that the virus successfully establishes in the midgut (Figure 2A points and lines). These findings are supported by the results of another modelling study that used a stochastic model of infection dynamics (Lord and Bonsall 2023).”*

*In discussion:*

*“Others have shown that stochastic individual-based models of the invasion process can be used to understand the dose-dependency in the probability a mosquito becomes infected (Lord and Bonsall 2023); interestingly, this other work also hints at there being a strong dose-dependency in dissemination.”*

*“Extending either our framework to model individual virus particles or extending other stochastic models (Lord and Bonsall 2023) to incorporate interactions with the basal lamina layer could help to understand whether purely random phenomena can explain why some mosquitoes become infectious and others do not under the same experimental conditions or whether other mechanisms, such as individual differences in the initial blood meal size, virus inoculum, or variation in immune response account for this difference.”*

General: I’d like to know why you didn’t go for a stochastic approach in the first place? In addition, why you didn’t mechanistically model cell infections which may have led to the model having a greater number of more biologically meaningful parameters (see comment on Line 76)?

*This is a great point, and it is why we devoted large sections of the discussion to the next obvious step: developing a more mechanistically inspired stochastic model. Ultimately, though, we think both stochastic and ODE-based models have merit. Here, we chose an ODE-based model because a) as we demonstrate, such a model can already explain key characteristics about the course of infection within mosquitoes, including how a second noninfectious bloodfeeding could influence dissemination rates and how sensitive such an effect might be to changes in the parameters governing the proliferation of the virus; b) some aspects of the mosquito-virus interactions remain poorly understood and, in some cases, phenomenological modelling allows us to sidestep these unknowns; and c) fitting stochastic models to data is inherently more expensive (our likelihood-based inference was already complex) or involves making approximations such as the use of Approximate Bayesian methods.*

Line 48: ‘*With the exception of a single study (21) measurements of dissemination represent the proportion of all blood-fed mosquitoes with disseminated infection*’ but in (22) Lequime et al (2020) they also report that ‘*systemic infection prevalences were measured by counting the number of mosquitoes with infected heads over the number of mosquitoes with an infected body*’ and Fig. 4 in this paper demonstrates the effect of virus dose and time on the proportion of infected mosquitoes with disseminated infection. So, there are actually two studies that do show the results in this way.

*Thank you for this reference. We have now amended the sentence referring to the single study to the following:*

*“While some studies measure dissemination as the proportion of all blood-fed mosquitoes with disseminated infection (Tesla et al. 2018; Lequieme et al. 2020), this makes it impossible to separate out the impact of changes to the bloodmeal titer on infection and subsequent dissemination. Thus we measured dissemination as the proportion of mosquitoes with infected midguts which have disseminated infection.”*

Line 49: In (21) - Tesla et al (2018), in the results it states in the results in section ‘*The effect of viral dose on ZIKV transmission efficiency’ ‘we did identify a significant interaction between ZIKV dose and days post-infection for both response variables (dissemination and transmission efficiency). This interaction demonstrates that variation in viral dose significantly affects the rate at which virus escapes the midgut and salivary gland barriers…’* Therefore, please remove ‘*with small sample size’* on line 49 as I don’t see how this is relevant, given they found a significant interaction between dose and time.

*Fair point; we have now removed any reference to the sample size.*

I would also suggest that it is possible from other studies to get the proportion of infected mosquitoes with disseminated infection just by swapping the denominator; it’s just not usually how these results are presented aside from in references 21 and 22. I think the more pertinent thing to highlight is perhaps how few studies carry out at least three virus doses and at least three time points to obtain data sufficient to quantity dose-time effects on probability of dissemination?

*Thank you for raising this point. We agree that sometimes sufficient information is present so that a reader could calculate the proportion of infected mosquitoes with disseminated infection; though often this information is encoded in graphs without numbers. Authors and readers often do, in our experience, however convolve the two presented quantities – the proportion of mosquitoes with disseminated infection and the proportion of infected mosquitoes with disseminated infection – and this is partly why we raised this point.*

*We agree with your point re: collecting more comprehensive data, however, and we have added this to the first paragraph of the discussion:*

*“In this study, we presented detailed data from laboratory experiments across multiple infection doses and multiple time points, which were then used to develop a mathematical model of viral population dynamics within mosquitoes.”*

Line 76: mechanistic model – only parts of it are mechanistic right? – it might be good to justify in the introduction or methods why you didn’t explicitly model virus-cell interactions?

*We agree that elements of the model are phenomenological in nature. But other parts are mechanistic in that they represent processes such as viral proliferation and spreading and the recovery of the basal lamina layer and its influence on midgut escape. We agree that modelling virus-cell interactions is important (and so are likely the within-cell signalling networks that support the mosquito immune response), and it is our intention to expand our work to consider these in further, stochastic and spatially resolved models of the infection process within the mosquito (building on our model and the methodology you report in your study).*

Line 78: ‘*Our results suggest that higher infectious doses hasten the rate at which mosquitoes become infectious’*. This has already been shown in 21 and 22, so perhaps frame the last paragraph of the introduction as a set of aims instead which explicitly shows how this study adds to the knowledge generated in 21 and 22? How is this study different? Does the mechanistic approach genuinely contribute more insight than statistical approaches?

*This is an excellent point, and we agree that our original framing was off. We have reframed this part of our introduction to read as follows:*

*“In this paper, we introduce a mechanistic model of within-mosquito viral dynamics and fit the model to experimental data collected by infecting Ae. aegypti mosquitoes with DENV. Despite being relatively simple, our model provides quantitative predictions which are consistent with our experimental data about how increasing the infection dose influences the rate at which mosquitoes become infectious -- a phenomenon that has been demonstrated in previous experimental studies (Tesla et al. 2018; Lequieme et al. 2020) and modelling work (Lord and Bonsall 2023). It also provides a framework that could be used to explore how perturbations to key mechanisms, for example, due to viral mutations or environmental fluctuations, may affect transmission rates. Importantly, our model shows that allowing the rate at which the virus can escape from the midgut to depend on the physical integrity of the encompassing basal lamina layer can explain why mosquitoes bloodfed on a second noninfectious bloodmeal may hasten dissemination rates -- a hypothesis previously suggested by Armstrong et al. (Armstrong et al. 2020). Simulations from our model indicate that there is a sweet spot in parameter space, where this double-feeding effect is strongest, so small changes in the parameter values may lead to substantial changes in the importance of repeated feeding on transmission. Our model is a step towards developing a higher fidelity model of viral infection dynamics within mosquitoes and, more generally, demonstrates the utility of collaborations between vector biologists and mathematical modellers in advancing understanding of within-vector viral dynamics.”*

Line 79: ‘*Our model also reveals a mechanism by which an initial infectious blood meal followed by a non-infectious blood meal can increase this rate, a characteristic of the experimental data we collected*…’. Again, unless this is the format requested by the journal, I think it would be better to outline the aims of the study. Here, after reading the method, I am not sure that the model ‘reveals’? It more appears that you have observed this phenomenon then developed a model that incorporates this with a term that accounts for slower rates with time post bloodmeal(s)?

*We agree. We have rephrased this as described in the response to your previous comment.*

Methods:

Line 180: What is the evidence that there is some maximum invasion rate?

*The mechanisms by which viruses invade cells are, to the best of our knowledge, poorly understood. However, to us, it seems reasonable to suppose there are limits on the rate of cell invasion of viruses: for example, if viruses must bind with cell-surface receptors before successful invasion occurs.*

Line 186: What is the evidence that the stretching of the midgut influences the rate at which virions invade midgut epithelial cells?

*Before a mosquito bloodfeeds, the mosquito lumen is a shape resembling an empty balloon. After a bloodmeal, the mosquito lumen takes on a shape more resembling that of a rugby ball (Kantor et al. 2018). To us, it seems reasonable to suppose that expansion of the lumen when a bloodmeal is imbibed then exposes more of the midgut epithelium to the virus – increasing the rate of viral invasion. To emphasise that this is a hypothesis rather than an experimentally known fact, we have amended the text around this description of the mathematical model to read:*

*“Stretching of this layer exposes a higher surface area of the epithelium to the lumen, since the shape of the lumen changes dramatically subsequent to bloodfeeding (Kantor et al. 2018), and we allow a faster rate of viral invasion to result through an increased rate of contact between virus and midgut epithelial cells. Since the mechanism by which viruses invade midgut cells remains, however, poorly understood, it would be useful to probe this hypothesis through future experimental work.”*

Line 228: without time series for individual specimens – but you do have how titer differs between specimens at a single time point and also the data on proportion infected by dose and time?

*Yes, we do have information on how measured titer differs across specimens at a single time point. But we cannot determine whether this variability is due to characteristics of the measurement process (e.g. idiosyncratic deviations between the actual and observed titer levels) or is due to inherent differences across individual mosquitoes (e.g. differences in parameters governing mosquito-virus interactions). To clarify, we have rephrased this sentence as:*

*“The dynamics are likely to be heterogeneous across specimens (Novelo et al. 2019), but without time series for individual specimens, it is not straightforward to separate variation in underlying dynamics from inherent variation due to imperfect measurement of viral titers.”*

Line 229: Measurement of what?

*We have now clarified this per the above change to this sentence.*

Line 230 to 234: Why were these methods not used in the current study?

*This is a good point. These new techniques produce measurements which are somewhat removed from the more direct measurements obtained from dissections, so we think that more work is required to clarify these relationships, and that this is best handled in future work. As such, we have amended our discussion to include the following:*

*“Alternative approaches which allow for a measure of individual-mosquito level infection dynamics by monitoring the level of virus in mosquito excreta or saliva that is expectorated during bloodfeeding show promise and further work is necessary to compare these techniques with the results obtained by dissection [Mayton et al. 2021…]”*

Line 250/ 314: With reference to S1A: this seems to indicate that the functions wouldn’t go to zero? Can you explain in the legend what ‘concentration’ represents – can this be related back to actual PFU/ml in experiments? It also seems that the function assumes that there will never be all mosquitoes infected irrespective of the virus concentration of the bloodmeal?

*This was a really useful comment, and we have changed our logistic function so that it behaves more mechanistically: yielding zero infection when zero concentration is given; and giving 100% infection at an infinite concentration. Here, concentration is parameterised in terms of 1/dilution and we avoid direct comparison with PFU/ml of the dose since we allow the initial dose to vary idiosyncratically across experiments (in our statistical model) and wish to avoid reference to a particular dilution.*

*The changes in this function mean that we have rerun all our results (adapting our priors so that they remain wide – see Figure S1); fortunately, this led to only minor changes in our figures. We thank the reviewer again for helping us to improve our study.*

Lines 249 – 268: For non-mathematicians would it be possible to explain in lay terms here how the logistic function is used with the ODE model to get results like that shown in Fig 2A – how you get variation in % midguts infected over time? It’s not clear to me, so perhaps it’s not clear to others.

*We thank the reviewer for their suggestion, and we agree this was not clear in our original manuscript. In the experiments performed, a specimen is deemed positive for infection if the measured viral titer exceeds a threshold value. This allows us to calculate the percentage of infected midguts (or legs) from our modelled results by calculating the probability that the measured value will exceed this threshold: this is given by eq. (8) for the midgut tissue; and by eq. (11) for the other tissues.*

*We have now added explanatory comments to figs. 2, 5, 6, S3 and S5 to explain the provenance of these % values.*

**Results**

Line 358 – would be better to be quantitative rather than ‘rapidly’ – is this seconds/ minutes/ hours/ days?

*We think that it is ok to keep the title of this section general since we provide references within it that suggest that clarify the speed (i.e. that infection occurs within hours of blood feeding).*

Line 364 – here it says that the first time point you have data for is 3 DPI so how can any assumptions be made about what is happening prior to this time point? Most of the blood meal will have been digested by day 3, therefore I would expect very little virus to be left in the luman after this point. Suggestion of infection prior to 3 DPI isn’t really rapid.

*We thank the reviewer for their comment. Whilst we do not have earlier dissection data, our model is able to probe before 3 DPI and suggests that infection occurs rapidly. The sentences prior to our statement about the rapidity of infection, we believe, clarify that we do not think that infection within 3 DPI is rapid – it is just that our results are supportive of those previously that suggest most infection occurs within hours of bloodfeeding. We have amended these sentences to make clear that it is previous work that informs 4-8 hours post-bloodfeeding being the appropriate timescale:*

*“It is thought that in order for an infection to occur, virus must invade the mosquito midgut epithelium prior to formation of the peritrophic matrix, which previous work suggests starts to form 4-8h post-feeding (Perrone et al. 1988). Our experimental data and modelling support this early viral invasion hypothesis.”*

Line 369 – and Fig2A – as per my comment in the method, because I’m not quite clear how the logistic function is linked to the ODE model, I can’t understand what is determining the rapid increase, then plateau of % of infected midguts, could there also not be a model structure where there is a much slower rise in the first 3 days? You don’t have data to support what is happening prior to 3 dpi. In the least perhaps in the Discussion describe what aspects of the model determine these early events and highlight the need for data from earlier time points to inform.

*We thank the reviewer for their comment. We have added the following to our methods to hopefully explain the link further:*

*“Note that eq. (8) combines both the logistic-type model given by eq. (5) for the presence/absence of infection and the ODE system solution to determine the probability that DENV titers exceed the threshold.”*

*It is correct that we do not have data before day 3, but we do have a series of experimental measurements taking place between 3-5 DPI across multiple concentrations (Fig 2A and Fig S3), which suggest that infection rates have plateaued by this time. This means that the rapid increase in infection rates must have happened well before this time, and this is what our model fits reflect, which coheres with previous experimental data (Perrone et al. 1988). As per your suggestion, we have added the following to our discussion:*

*“In future work, we plan to probe the initial stages of midgut infection by collecting experimental data from early on during the midgut infection in order to verify our modelled results in this period.”*

There are some lines that are better off in the Introduction or Discussion e.g. line 361, 368-372, 385-393, 407 – 412, 426 – 429, 432 – 437, 462 – 467, 496 - 509. A lot of these read more like discussion, so please just report your results here and leave comparison with other studies to the discussion section. This will make the results a lot clearer

*We thank the reviewer for their suggestion. We suppose this comes down to a question of style, and it seems that this journal allows results sections of both types: either a strictly results-only section (e.g.: https://journals.plos.org/plospathogens/article?id=10.1371/journal.ppat.1011685); or a more discursive style (e.g. https://journals.plos.org/plospathogens/article?id=10.1371/journal.ppat.1011679). We here prefer to interweave our results in with their wider context since the journal focuses on the biology of pathogens rather than purely computational modelling, and we believe our narrative may be more interesting and easier to follow for a computationally naive audience.*

Line 381: Rather than describing what the plot is please describe the results in the main text and just refer to the figure.

*We think this comes down to style, and we would prefer to keep to the format we have currently.*

Line 381 and Fig 2B: it looks like the fitted model plateaus at c. 25% below a concentration of 0.03 – this to me doesn’t make biological sense and indeed doesn’t seem to fit well to the lowest two virus concentrations. Can you explain why it plateaus here rather than going to zero as virus concentration declines to zero?

*This is an astute point and we have modified our choice of logistic function and refit the model so that it goes through 0% when the concentration is 0. Our new Figure 2B provides a closer fit to the experimental data.*

Line 422: ‘Faster growth in the level of virus’ – do you mean: i) in the midgut or other tissues; and ii) higher virus replication rate or earlier escape leads to earlier replication in other tissues which leads to higher viral titres?

*We have changed the sentence to avoid this ambiguity:*

*“Faster growth in viral titer in the legs means that disseminated infection is detected earlier in a higher proportion of specimens (see Figure 5B).”*

Line 423: I don’t see how the earlier detection of dissemination leads to less time required for dissemination for higher initial doses – e.g. this is not a consequence of what is detailed in the prior sentence.

*Thank you. We have rephrased this to:*

*“A consequence of this is that, on average, less time is required for detectable disseminated infection to occur for higher initial doses (Figure 5C).”*

Line 425: ‘…extrapolating…’ – is this not a benefit of a mechanistic approach that extrapolation should give something insightful, even if we have to keep model assumptions and alternative models in mind (as per the findings in Fig 2A)?

*You are right that it is a benefit but we think it reasonable to point a reader to this extrapolation here.*

Line 430: Rather than ‘mutations’ I would state the parameters in your model that the midgut and dissemination rates are sensitive to in the subtitle. I also guess you mean ‘mutations affecting’. By doing this you also get away from stating the obvious – of course midgut invasion will be sensitive to mutations that affect midgut colonisation; this is a circular argument.

*We thank the reviewer for pointing out the typo – this is corrected. We would prefer to keep the focus on the biology rather than our choice of mathematical notation, and so would prefer to avoid explicitly including the parameters in the subtitle. Though, we agree with the reviewer’s sentiment and have changed the subtitle to read:*

*“Midgut invasion and dissemination rates are sensitive to fluctuations in parameters representing viral establishment and less sensitive to changes in the rate of viral digestion in the lumen”*

Line 439: you say flavivirus here, but CHIKV is an alphavirus – why not just mosquito-borne virus?

*Thank you. We have changed this to:*

*“We now use our mathematical model to explore how changes to three parameters which characterize the ability of an arbovirus to invade the midgut affect midgut infection and dissemination.”*

*We have also changed the start of our abstract to reflect that our model is more generally relevant:*

*“Arboviruses can emerge rapidly and cause explosive epidemics of severe disease. Some of the most epidemiologically important arboviruses, including dengue virus (DENV), Zika virus (ZIKV), Chikungunya (CHIKV), and yellow fever virus (YFV), are transmitted by Aedes mosquitoes, most notably Aedes aegypti and Aedes albopictus. After a mosquito blood feeds on an infected host, virus enters the midgut and infects the midgut epithelium. The virus must then overcome a series of barriers before reaching the mosquito saliva and being transmitted to a new host. The virus must escape from the midgut (known as the midgut escape barrier; MEB), which is thought to be mediated by transient changes in the permeability of the midgut-surrounding basal lamina layer (BL) following blood feeding. Here, we present a mathematical model of the within-mosquito population dynamics of DENV (as a model system for mosquito-borne viruses more generally) that includes the interaction of the midgut and BL which can account for the MEB.”*

Lines 438 – 446: This should probably go in the methods.

*Thank you although we think this is fine here as otherwise we are asking the reader to hold quite a few details in their head.*

Lines 447 – 449: Just state what the results are rather than stating what you plot (e.g. something like ‘*Simulations (Fig S4) show that a two-order*…’

*We think this comes down to style, and we would prefer to keep to the format we have currently.*

**Discussion**

Line 554 – 556: ‘crucial to gauge transmission intensity’ – here if you mean force of infection during an outbreak, I suggest that this is not the case, this can be determined from age-seroprevalence curves, measuring cases per unit time etc. In addition, ‘develop effective public health interventions’ is also too generic – I would argue it’s not essential for human vaccine development or necessarily vector control. Can you be more specific here.

*We agree. We have rephrased this first discussion sentence to read:*

*“Understanding the biological mechanisms which influence the life cycle of viruses within their mosquito vectors is crucial for developing public health interventions which aim to target these life stages.”*

Line 561: I would stick to describing the model parameters rather than talking about mutations here and be more specific.

*We agree and have changed this sentence to read:*

*“Our work also hints that the magnitude of this double-feeding effect may be particularly sensitive to changes in the rate of viral proliferation in the midgut, which suggests the effect size may differ across arbovirus-mosquito combinations.”*

Line 562: ‘series of models at different scales’ – can you be more specific about what you have in mind here? Do you mean coupling within-mosquito with population-level or within-cell and within-mosquito? Without more specific detail the statement is a bit empty.

*We have rephrased this to add more detail:*

*“Our model represents a step towards what we envisage as a series of mechanistic models at different scales -- from the subcellular to the tissue level -- which can be used as tools for understanding this complex system.”*

Line 577: I would suggest that they don’t necessarily avoid the bottlenecks, indeed there is some evidence that they may be advantageous (see discussion in our Plos Comp Bio paper).

*We thank the reviewer for pointing us towards their discussion. We have reformulated this section to read:*

*“Whether wild populations avoid these bottlenecks is unclear since infectious doses in the wild are thought to be considerably lower than those required in the laboratory to produce successful infection (Turell et al. 1988l; Weaver et al. 2021), and midgut infection has been demonstrated to present a particularly strong barrier at low infection doses (Forrester et al. 2012). Some have even suggested that these bottlenecks may be evolutionarily advantageous by selecting against defective virus particles early on during infection (Lord and Bonsall 2023).”*

Line 659: I would omit this sentence as I don’t think it’s necessary and you are assuming that biologists don’t think quantitatively. This is way too generalising of biologists!

*We agree and have removed this inflammatory line.*

**Reviewer #3**

The paper presents a novel mechanistic within-vector model of dengue infection in Aedes mosquitoes. The model is fitted to data on dengue infection, including single and double blood feeds. The topic is very relevant and not extensively examined for viral infections. Overall, the paper is well written, with clear justification of modeling choices, and acknowledgement of limitations. While the model is simplistic, it is an excellent first step with a nice road map of ways to improve the model (e.g. stochasticity). In particular, the careful attention to detail on the model formulation, providing all the necessary components for replication, is rare and is much appreciated.

*We thank the reviewer for their summation; we’re particularly pleased to hear that they valued the attempts we went through to ensure replicability of this work.*

**Major comments**

No major issues.

**Minor comments**

It is unusual to call the model “time-dependent” as done in the methods and discussion. The equations are autonomous, with no explicit dependence on time. It would be more accurate to call the model a system of autonomous ODEs and remove “time-dependent”

*Thank you. Our ODEs do not contain time other than as the independent variable. As such, we have changed their description to “autonomous ODEs” throughout the manuscript.*

- Why is a Hill coefficient of 2 chosen for the rate that the virus invades the midgut? This typically indicates cooperation. This is one of the few modeling choices not discussed.

*We thank the reviewer for this astute comment, and we have now changed this coefficient to 1 as, whilst there is some evidence of cooperative effects (e.g. multiple virions invading a cell could overwhelm immune responses), we agree that it is more parsimonious to assume noncooperativity. This change has meant that we have rerun our analyses which has resulted in minor changes to the results: i.e. only quantitative rather than qualitative shifts.*

- In general, all figures could do with larger font.

*We have changed font sizes across our figures.*

- In Figures 2 and 3, the colors can be hard to distinguish (especially for the points). Consider using different marker symbols.

*We thank the reviewer for their suggestion; we have chosen different marker symbols for the different concentrations in these figures.*

- In Figure 5 B and C, the font is small to the point of being difficult to read.

*Thank you. We have changed this.*

- In Figures 6, S1, S4, S5, S6, S7, write out what DPI means.

*Thank you. We have changed this.*

- In Figure 7, label the axis with words in addition to symbols. The symbols are not mentioned in the caption either.

*This is a good point; we have changed the caption to reflect this (since the symbols and descriptions were too long to include in the axis labels).*

- In Figure S2, what does *** indicate?

*This is an excellent point. These indicate the statistical significance of the estimated correlation coefficients. We have now added this information to the figure caption.*

- The final paragraph seems out of place. While this reviewer agrees with the sentiment, it’s not clear it’s appropriate at this point in the manuscript.

Thank you. We have changed it so that it now reads:

*“Vector biologists have made great leaps in our understanding of the predominant mechanisms underpinning the life stages of pathogens within mosquitoes. However, due to the complexity of the vector-pathogen system and the interaction between many different cellular and subcellular elements, unpacking the impact of different processes often requires quantitative models. Thus, we argue, the process of learning vector and pathogen biology can be greatly enhanced through collaborations between biologists and mathematicians.”*
