# Supplementary material for: Investigating the dose-dependency of the midgut escape barrier using a mechanistic model of within-mosquito dengue virus population dynamics
Source: PLoS Pathog. 2024 Apr 1;20(4):e1011975. doi: 10.1371/journal.ppat.1011975 (PMC11008821; doi:10.1371/journal.ppat.1011975)
Supplement: Attachment — Submitted filename: plos_path_response_2nd_review.docx [file ppat.1011975.s015.docx]

Dear Editors,

We thank you again for handling our review and the reviewers for their useful feedback.

Below we show our response to reviewer 2’s comments since they were the only reviewer requesting changes.

We confirm that we have used the PACE tool to check the requirements of the figures for the journal. All of our data and code are freely available through the open-source repository named in the manuscript.

Alongside this letter, we make two versions of the manuscript available: one with the newest change highlighted and another without this single change to the text. We hope you will find our new manuscript suitable for publication and thank you again for considering it.

Yours sincerely,

Ben (on behalf of coauthors)

### Reviewer #2

I still think the manuscript could be made somewhat more concise by restricting the results section just to the results and therefore easier to digest, but accept that this is just a difference in opinion concerning style.

*We thank the reviewer for their comment and accept that this is down to a difference in style.*

From line 48 statement is still not correct:
While some studies measure dissemination as the proportion of all blood-fed mosquitoes with disseminated infection [21, 22], this makes it impossible to separate out the impact of changes to the bloodmeal titer on infection and subsequent dissemination. Thus we measured dissemination as the proportion of mosquitoes with infected midguts which have disseminated infection.

Both studies measure and present results on the proportion of infected mosquitoes that have a disseminated infection, which makes it possible to separate out the impact of changes.

*We thank the reviewer for pointing this out; we have now changed these to read:*

*“*Most studies measure dissemination as the proportion of all blood-fed mosquitoes with disseminated infection making it impossible to separate the impact of changes to the bloodmeal titer on infection and subsequent dissemination. Some studies do, however, measure dissemination as the proportion of mosquitoes with infected midguts which have disseminated infection [21, 22], and here we follow suit.”

Fig 1. D. ‘other tissues’ should be h(t) not m(t)

*Great spot, thank you – we have now changed this.*
